# Alternative methods of globotrioside production using Vero cells: a microcarrier system procedure

**DOI:** 10.1186/1752-153X-1-26

**Published:** 2007-11-05

**Authors:** Atsushi Miyagawa, Maria Carmelita Z Kasuya, Kenichi Hatanaka

**Affiliations:** 1 Center for Collaborative Research, The University of Tokyo, 4-6-1 Komaba, Meguro-ku, Tokyo 153-8505, Japan; 2 Institute of Industrial Science, The University of Tokyo, 4-6-1 Komaba, Meguro-ku, Tokyo 153-8505, Japan; 3 Research and Development Division, Japan Chemical Innovation Institute 1-3-5, Jimbocho, Chiyoda-ku, Tokyo 101-0051, Japan

## Abstract

**Background:**

Glycolipids are one component of cell membranes, and are found most prevalently at the surface of the plasma membrane. Animal cells take in amphipathic glycosides, which are later glycosylated after assimilation in biosynthetic pathways. Gycosylated glycosides are released outside of cells to the surrounding culture medium. This represents an accessible method of obtaining complex glycosides.

**Results:**

Vero cells are sensitive to Shiga toxins and are known to express the glycosides globotriaosyl ceramide (Gb3) and globotetraosyl ceramide (Gb4) on the surface of the plasma membrane. By administering amphipathic lactosides to Vero cells, the above mentioned glycolipids could be produced by the action of cellular enzymes. In our study, the optimum conditions (seeded cell number, incubated time period, 12-azidododecyl lactoside concentration and medium volume) for the production of Gb3 analogue were investigated. The 87.9 μg/100 mm dish (11.7 % yield) Gb3 analogue was produced under appropriate conditions. The large-scale culture of Vero cells using a microcarrier culture method with repetitions produced about 30 mg of the Gb3 analogue.

**Conclusion:**

The mass production of glycosides in Vero cells was carried out on a microcarrier with repeated administration of 12-azidododecyl lactoside. The results indicated that the use of both a microcarrier culture and repetition were highly effective in the production of Gb3, Gb4 and sialyl lactoside (GM3) type-oligosaccharides.

## Background

Glycolipids are one component of cell membranes, and are found most prevalently at the surface of the plasma membrane. They serve as signal molecules, being recognized by proteins such as lectins, bacterial toxins and viruses [1].

Glycolipids are composed of a ceramide and a sugar moiety synthesized in the endoplasmic reticulum (ER) and Golgi apparatus, respectively. Lactosyl ceramide is one precursor of complex glycolipids and can be glycosylated in the Golgi. Glycolipids prepared biosynthetically are then transported to the plasma membrane where they remain as components of cell membrane [2,3]. These pathways have been utilized to produce oligosaccharides using cellular enzymes or recombinant cells [4-6].

The commonest method of preparing glycosides is by chemical synthesis. However, many reports show that this preparation of glycosides is difficult, requiring several reaction steps and control of stereoselectivity of the glycosyl linkage. [7,8].

B16 melanoma cells strongly express sialyl lactosyl ceramide (GM3) at the surface of the plasma membrane. It has been reported that hexadecyl β-lactoside is sialylated in B16 cells. Therefore, B16 cells take in amphipathic lactosides dissolved in the culture medium and sialylated them, before releasing them into the surrounding culture medium. This method for the production of glycolipid analogues can be applied to various cells and alkyl glycosides [9,10].

When amphipathic glycosides with different sugar units are taken into a cell, the glycosides are further glycosylated by a variety of cellular enzymes resulting in various oligosaccharides. To ensure glycosylation of amphipathic glycosides, a series of requirements must be satisfied: First, the glycoside must be taken into the cell. Here the length of the alkyl chain of the aglycon is important; glycosides with C8 alkyl chains are taken in by B16 cells poorly, while those with C12 or C16 alkyl chains enter more efficiently. Second, the glycoside should be recognized by cellular enzymes. Third, the glycosylated product should be released to the culture medium; the elongated glycosides with C16 alkyl chains have been shown to remain mostly in the B16 cell, whilst those with C12 chains are released into culture medium [11].

In this study, we report the glycosylation of dodecyl lactoside or 12-azidododecyl lactoside in Vero cells. The production of the globotriaosyl ceramide (Gb3) analogue was optimized using appropriate conditions such as seeded cell number, concentration of the lactoside, time of incubation and culture medium volume. The administration of the lactoside and harvesting of glycosylated lactoside were repeated every 24 to 72 hours for twelve days. Because obtaining glycosylated product in large amounts on culture dishes is tedious, the efficient mass production of Gb3 analogue was accomplished using Vero cells cultured on microcarriers in spinner flasks. As compared to existing methods of production of Gb3 analogues, our method is fast and convenient, and does not require the use of expensive enzymes and glycosyl donor. In addition, controlling the stereoselectivity of the product is not a problem.

## Results and discussion

### Chemical synthesis of amphipathic glycosides

**Scheme 1:**
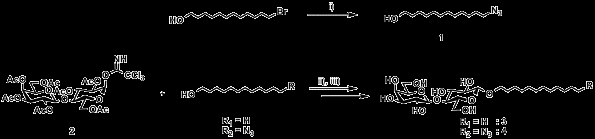
Synthesis of amphipathic lactosides. i) NaN_3_, DMSO, 80°C. ii) BF_3_·Et_2_O, ClCH_2_CH_2_Cl, 0°C. iii) NaOMe, MeOH, r.t.

Dodecyl lactoside is the standard used to investigate glycosylation in cells. We prepared dodecyl β-acetyl lactoside by reacting acetyl lactosyl imidate with dodecane-1-ol using BF_3_·Et_2_O (scheme 1). The dodecyl β-acetyl lactoside was then deacetylated with NaOMe in MeOH, with the amphipathic glycoside, dodecyl β-lactoside, obtained in a 51.6% yield (2 steps).

We also synthesized 12-azidododecyl lactoside. 12-Bromo dodecane-1-ol was reacted with sodium azide in DMSO yielding 12-azidododecane-1-ol, which was then glycosylated with lactosyl imidate by the same procedure, before being deacetylated to give 12-azido dodecyl lactoside in 61.3% yield (3 steps). The product has the azido group at the ω-position of the alkyl chain, which can be easily converted to an amino group for further utilization.

### Glycosylation of dodecyl lactoside by Vero cells

**Figure 1:**
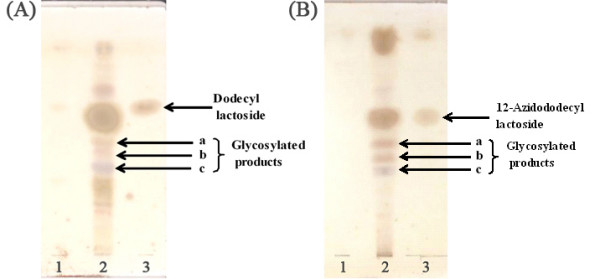
HPTLC results. (A) Glycolipids after incubation of Vero cells with dodecyl lactoside. Lane 1, glycolipids extracted from cell homogenate; Lane 2, glycolipids extracted from medium; Lane 3, dodecyl lactoside. (B) Glycolipids after incubation of Vero cells with 12-azidododecyl lactoside. Lane 1, glycolipids extracted from cell homogenate; Lane 2, glycolipids extracted from medium; Lane 3, 12-azidododecyl lactoside.

To investigate how the amphiphatic lactoside is glycosylated, dodecyl lactoside was incubated with Vero cells. After incubation, the glycolipids – which were collected from the culture medium and cell homogenates – were analyzed by HPTLC. As shown in Figure 1 (A), three new bands, which were not obtained in the control, were observed. It was thought likely that these corresponded to Gb3, Gb4 and GM3 type oligosaccharides, owing to these being known to occur naturally in Vero cells [12-15].

To determine the structure of the glycosylated sugar moiety, the glycosylated lactosides were scraped from the HPTLC plates, collected and extracted. Owing to the small quantities of collected glycosides NMR spectra could not be measured; analysis was thus carried out by ESI-MS. The mass spectra results showed peaks at m/z 533.4, 695.1, 898.5 and 987.7 corresponding to [dodecyl lactoside + Na]^+^, [dodecyl Gb3 + Na]^+^, [dodecyl Gb4 + Na]^+ ^and [dodecyl GM3 + Na]^+^, respectively. These results are consistent with the glycosides anticipated from HPTLC analyses. The three new bands were thus assumed to be analogues of Gb3 (galactosylated lactoside derivative), Gb4 and GM3. These oligosaccharides were produced by only one cell type, therefore, different kinds of oligosaccharides can be produced using various cells. This method of administering the glycosides to the cells is also effective in biocombinatorial synthesis for the construction of an oligosaccharide library [16].

In the same manner, 12-azidododecyl lactoside was incubated with Vero cells and the glycosides in the culture medium were developed on HPTLC. From HPTLC analysis, three new bands were observed (at same positions observed for dodecyl lactoside) and corresponding to Gb3, Gb4 and GM3.

### Influence of seeded cell number

**Figure 2:**
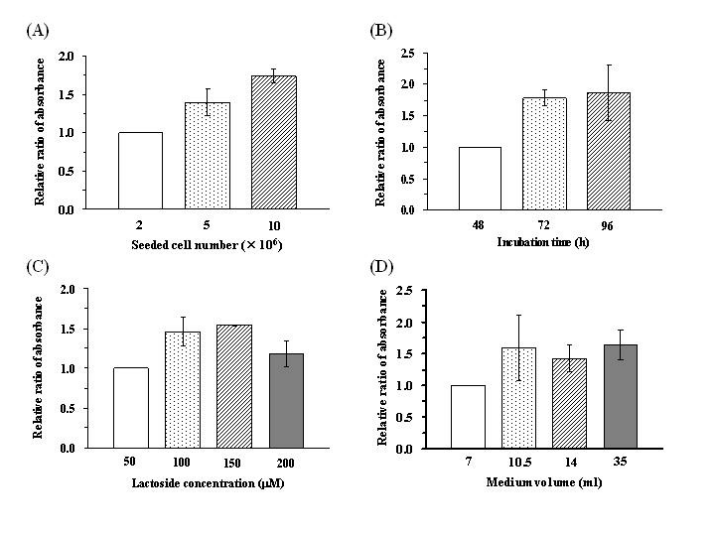
Influence of cell culture conditions. Relative ratio of the amount of product in different (A) seeded cell numbers; (B) incubation times; (C) lactoside concentrations; and (D) medium volumes.

To evaluate the influence of seeded cell number on the yield of glycosylated lactoside, 2 × 10^6^, 5 × 10^6 ^and 10 × 10^6 ^Vero cells were seeded on 100 mm dishes. The seeded cells were incubated for 24 h in a medium containing 10% FBS before being incubated in a medium containing 50 μM of 12-azidododecyl lactoside for a further 48 h. Quantification of the amount of glycosylated product with a densitometer revealed that the relative ratio of the absorbances of the galactosylated lactoside bands was 1.00 (2 × 10^6 ^cells): 1.39 (5 × 10^6 ^cells): 1.74 (10 × 10^6 ^cells) as shown in Figure 2 (A). Therefore, the quantity of glycosylated product increased with increasing seeded cell number, with 2 × 10^6 ^cells most efficient for the quantity of product obtained per-cell.

### Influence of the incubation time

To evaluate the influence of incubation time on the yield of glycosylated lactoside, 2 × 10^6 ^Vero cells were seeded on 100 mm dishes. The cells were incubated in a medium containing 10% FBS for 24 h, before further incubation for 48, 72 and 96 h in a medium containing 50 μM 12-azidododecyl lactoside. Analysis of the amount of glycosylated product with a densitometer revealed that the relative ratio of the absorbances of the galactosylated lactoside bands was 1.00 (48 h): 1.78 (72 h): 1.84 (96 h) as shown in Figure 2 (B). An incubation time of 72 h was most efficient in terms of the amount of product obtained as a function of time.

### Influence of the concentration of 12-azidododecyl lactoside

To evaluate the influence of the concentration of 12-azido dodecyl lactoside on the yield of glycosylated lactoside, 2 × 10^6 ^Vero cells were seeded on 100 mm dishes and incubated in a medium containing 10% FBS for 24 h. Subsequently, the dishes were incubated in media containing 50, 100, 150 and 200 μM of 12-azidododecyl lactoside for 48 h. Quantification of the amount of glycosylated product with a densitometer revealed that the relative ratio of the absorbances of galactosylated lactoside band was 1.00 (50 μM): 1.46 (100 μM): 1.54 (150 μM): 1.17 (200 μM) as shown in Figure 2 (C). The largest amount of galactosylated lactoside was obtained from the incubation with 150 μM of lactoside, while cytotoxicity was observed for the 200 μM medium (See additional file 1: Figure 4). The medium containing 50 μM of lactoside was seen to be the most efficient in terms of the yield of galactosylated lactoside.

### Influence of medium volume

To evaluate the influence of media volume on the yield of glycosylated lactoside, 5 × 10^6 ^Vero cells were seeded on 100 mm dishes. The cells were incubated in a medium containing 10% FBS for 24 h, before incubation for 48 h in 7.0, 10.5, 14 and 35 ml of media containing 50 μM 12-azidododecyl lactoside. Analysis with a densitometer of the quantity of glycosylated product revealed that the relative ratio of the absorbances of galactosylated lactoside bands was 1.00 (7 ml): 1.59 (10.5 ml): 1.42 (14 ml): 1.63 (35 ml) as shown in Figure 2 (D). Even if the volume of the medium were increased, the yields of galactosylated lactoside were almost similar to those obtained from 10.5 ml. Therefore, the 10.5 ml medium was deemed most suitable for the production of glycoside.

### The quantity of galactosylated lactoside obtained under optimum conditions

We selected the following conditions: seeded cell number of 5 × 10^6 ^or 10 × 10^6 ^cells; 72 h incubation period; lactoside concentration of 100 or 150 μM; and medium volume of 10.5 ml. Under these conditions, Vero cells were seeded on 100 mm dishes and incubated in media containing lactoside (See additional file 2: Figure 5). The results showed that the highest yield of galactosylated lactoside obtained was 87.9 μg/dish (11.7 %). From the results, the optimum set of conditions were seen to be a 72 h incubation period, using 5 × 10^6 ^cells in the presence of 100 μM lactoside and 10.5 ml of medium solution.

### Repeated administration and harvest

**Figure 3:**
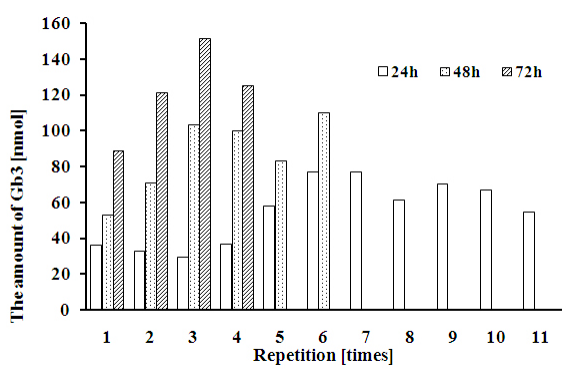
Galactosylated lactoside (Gb3) produced after repeated administration of 12-azidododecyl lactoside in Vero cells with harvest of incubated medium at repeated time periods.

After the solution containing galactosylated lactoside derivative was harvested from the medium of Vero cells, the lactoside-containing medium was successively added and incubated. The concentration of lactoside in the media was 100 μM, with the administration and harvest carried out every 24, 48 or 72 h. The time period of the cell culture was twelve days, with the amounts of galactosylated lactoside from each of harvested media quantified using densitometer (Figure 3).

In the case of the 24, 48 and 72 h cycles, the total quantities of galactosylated lactoside obtained were 430 μg (602 nmol, 5.21%), 371 μg (520 nmol, 8.25%) and 347 μg (486 nmol, 11.6%), respectively.

These results indicated that Vero cells are suitable for continuous culture over long, repeated time periods and are useful for the mass production of glycosides.

### Large-scale production of galactosylated lactoside

For the production of large amounts of galactosylated lactoside, Vero cells were seeded on a microcarrier in a spinner flask and cultured for 2 weeks [17,18]. The concentration of lactoside was 100 μM and repetition cycles lasted 48 or 72 h. The results showed that a 48 h repetition time frame (rather than 72 h) was preferable, with Gb3 quantities obtained being 4.7 μg/ml 3.3 μg/ml for 48 and 72 h, respectively. Finally, Vero cells on a microcarrier in 4 litre spinner flask were incubated for 48 h, with the administration and harvest repeated 5 times. The total amount of galactosylated lactoside obtained was 30.2 mg (42.4 μmol, ^1^H NMR spectrum: H-1, J_1,2 _= 7.8; H-1', J_1',2_' = 7.2; H-1", J_1",2" _= 4.2).

## Conclusion

We have shown that dodecyl lactoside and 12-azidododecyl lactoside were taken into Vero cells, glycosylated by cellular enzymes, and released as glycosylated lactosides into the surrounding medium. The glycosylated lactosides were identified as Gb3, Gb4 and GM3-type oligosaccharides. The optimum conditions for the production of glycosylated lactosides were determined (seeded cell number of 5 × 10^6^; 72 h incubation period; lactoside concentration of 100 μM; medium volume of 10.5 ml) with galactosylated lactoside obtained in 11.6% yield. The lactoside was repeatedly administered and harvested, continued for twelve days. The large-scale production of glycosides in Vero cells was carried out on a microcarrier, resulting in the production of about 30 mg of product. Results indicated that both a microcarrier culture and repetition were highly effective for the production of glycosides, such as Gb3, Gb4 and GM3 type oligosaccharides.

## Experimental

### General methods

^1^H NMR spectra were recorded at 600 MHz using a Jeol ECP-600 spectrometer in chloroform-*d *or deuterium oxide. ^13^C NMR spectra were recorded at 150.9 MHz with the same instrument. Tetramethylsilane (TMS) was used as the internal standard. Assignments in the NMR spectra were made by first-order analysis of spectra, and supported by correlation spectroscopy and heteronuclear chemical shift correlation. Reactions were monitored by thin-layer chromatography (TLC) on a precoated plate of silica gel 60 F254 (layer thickness, 0.25 mm; E. Merk, Darmstadt, Germany). TLC sheets were dipped in (a) a solution of 85:10:5 (v/v/v)-methanol-*p*-anisaldehyde-concentrated sulfuric acid and heated for a few minutes (for carbohydrates). Column chromatography was performed on silica gel (Silica Gel 60; 40–63 mm, E. Merck, or Silica Gel 60, spherical neutral; 40–100 mm, E. Merck).

### Synthesis of amphipathic glycosides

#### 12-azidodecane-1-ol (1)

To a solution of 12-bromo dodecane-1-ol (12.6 g, 47.4 mmol) indimethyl sulfoxide (200 ml) was added sodium azide (15.4 g, 237 mmol), with the solution stirred at 80°C for 1 week. The mixture was then extracted with ethyl acetate, washed with water three times, dried over Na_2_SO_4_, and filtered, with the filtrate evaporated *in vacuo*. Purification by silica gel column chromatography with 10:1 (v/v) of hexane:ethyl acetate was carried out on the residue yielding crude **1 **(11.5 g):^1^H NMR (CDCl_3_) δ 3.61 (t, 2H, -CH_2_-O), 3.24 (t, 2H, -CH_2_-N_3_), 1.57 (m, 4H, -CH_2_-C-O, -CH_2_-C-N_3_), 1.27 (m, 16H, -CH_2_-).

#### Dodecyl β-lactoside (3)

Compound 3 was prepared by the method described in the literature [1,6,19]: ^1^H NMR (H_2_O) δ 4.19 (d, 1H, J_1',2' _= 6.1, H-1'), 4.16 (d, 1H, J_1,2 _= 7.7, H-1), 3.73 (br-d, 1H, -O-CH-C-), 3.37 (m, 1H, H-2'), 2.99 (br-t, 1H, H-2), 3.37 (br-d, 1H, -O-CH-C-), 1.50 (s, 2H, -O-C-CH_2_-), 1.23 (s, 20H, -CH_2_-), 0.85 (s, 3H, -CH_3_).

#### 12-Azidododecyl β-lactoside (4)

Compound 4 was prepared by the method described in the literature [1,6,19]: ^1^H NMR (H_2_O) δ 4.87 (d, 1H, J_1',2' _= 7.7, H-1'), 4.76 (d, 1H, J_1,2 _= 7.7, H-1), 2.00 (s, 4H, -O-C-CH_2_-, -CH_2_-C-N_3_), 1.77 (s, 18H, -CH_2_-).

### Cell culture

Vero Cells were obtained from Riken Cell Bank (Tsukuba, Japan). DMEM/F12 was purchased from Sigma and fetal bovine serum (FBS) was purchased from JRH Biosciences. Insulin-transferrin-sodium selenite-X (ITSX) was purchased from GIBCO. Sep-Pak C18 was purchased from Waters. HPTLC (silica gel 60) plate (E. Merck, Darmstadt, Germany) was used. The quantification of glycosylated products was carried out with CS-9300PC dual-wavelength flyingspot scanning densitometer (SHIMADZU).

Vero cells were cultured in DMEM/F12 supplemented with 10% FBS, detached through application of 0.25% trypsin/EDTA and maintained in humidified atmosphere of 5% CO_2 _at 37°C. Cells were cultured in 100 mm dishes. The lactosides were dissolved in Me_2_SO to an initial concentration of 50 mM.

### Production of elongated glycolipids by Vero cell

2 × 10^6^, 5 × 10^6 ^or 10 × 10^6 ^Vero cells were seeded on 100 mm culture dishes containing 7, 10.5, 14 or 35 ml of medium (DMEM/F12 containing 10% FBS) and incubated for 24 h. The cells were then washed with the medium (DMEM/F12 containing 1% ITSX), and incubated with 50, 100, 150 or 200 μM lactoside. After incubation at 37°C for 48, 72 or 96 h, the culture media were collected and the cells were washed with PBS (-), harvested with 0.25% EDTA in PBS (-), and the suspension centrifuged at 1000 rpm for 5 min. Lipid extraction was carried out according to the literature [20]. Lipids from the cell homogenate and culture medium fractions were analyzed by HPTLC with 5:4:1 CHCl_3_-MeOH-0.25% aq KCl as developing solvent. For identification of elongated glycosides, the bands of products were scraped and the products extracted with methanol. The solutions were then concentrated and the molecular weights measured using ESI-MS (Bruker Daltonics Inc., Esquire 6000). For the quantification of elongated glycosides, HPTLC plates were sprayed with resorcinol and heated to detect the separated glycolipids. The bands of glycolipids were measured, absorbance (410 nm), using a densitometer and quantified by applying a standard curve of five concentrations of hexenyl Gb3.

### Microcarrier culture

5 g of CELL YARD™ beads (Pentax Co.) were equilibrated in 100 ml medium and placed in a spinner flask (Shibata scientific technology ltd). 5 × 10^7 ^cells were seeded on the beads in the flask. The suspension was stirred for 1 minute at 37 rpm every hour. After 4 h, a medium containing 10% FBS was added and stirred continually for 2 days at 37 rpm, 37°C. The medium was then changed to one containing 1% ITSX and 100 μM of the 12-azidododecyl lactoside and incubation continued. The harvest and administration were carried out repeatedly and the collected media were treated according to the same method as described earlier.

The glycolipids could be extracted and purified from the collected culture media by reversed phase column chromatography (octadecyl) using methanol/water (gradient) as eluent. A more detailed discussion of the purification method employed in this research will be reported elsewhere by Kato *et al *[21].

## Authors' contributions

Dr. AM. participated in the design of the study and carried out all procedures. Dr. MK. helped in design and coordination, and the drafting of the manuscript. This project was based on the ideas of Dr. KH. and carried out with his guidance and consultation.

## Supplementary Material

Additional file 1 Vero cells with azidododecyl lactoside after incubation. The data provided represent the cytotoxicity of azidododecyl lactoside for Vero cells. Click here for file

Additional file 2 HPTLC results. The data provided represent the production of the glycosides under optimized conditions. Click here for file
