# Supplementary figures and images for: Alternative methods of globotrioside production using Vero cells: a microcarrier system procedure
Source: Chem Cent J. 2007 Nov 5;1:26. doi: 10.1186/1752-153X-1-26 (PMC2213644; doi:10.1186/1752-153X-1-26)

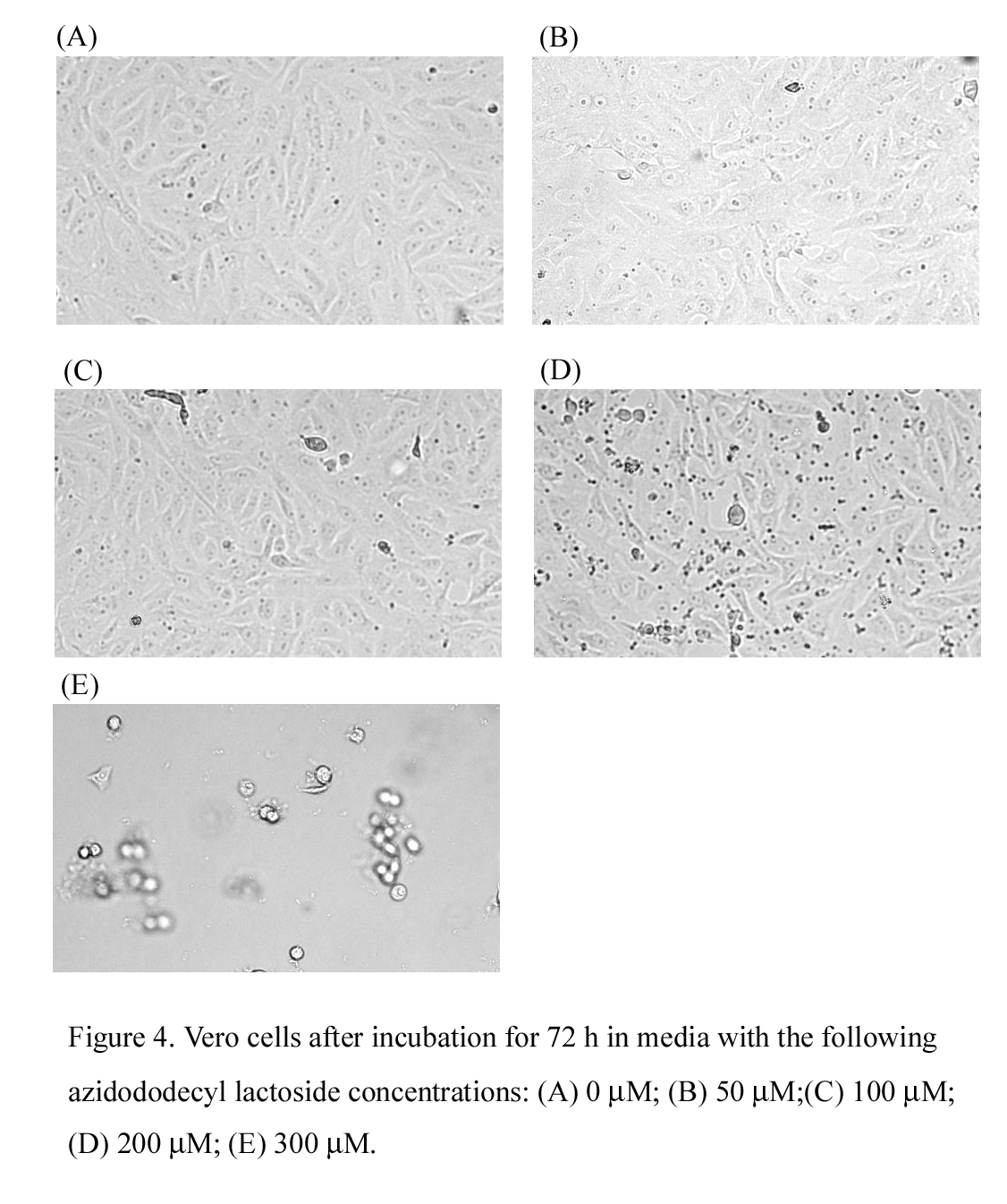

Supplement: Additional file 1 — Vero cells with azidododecyl lactoside after incubation. The data provided represent the cytotoxicity of azidododecyl lactoside for Vero cells. [file 1752-153X-1-26-S1.jpeg]

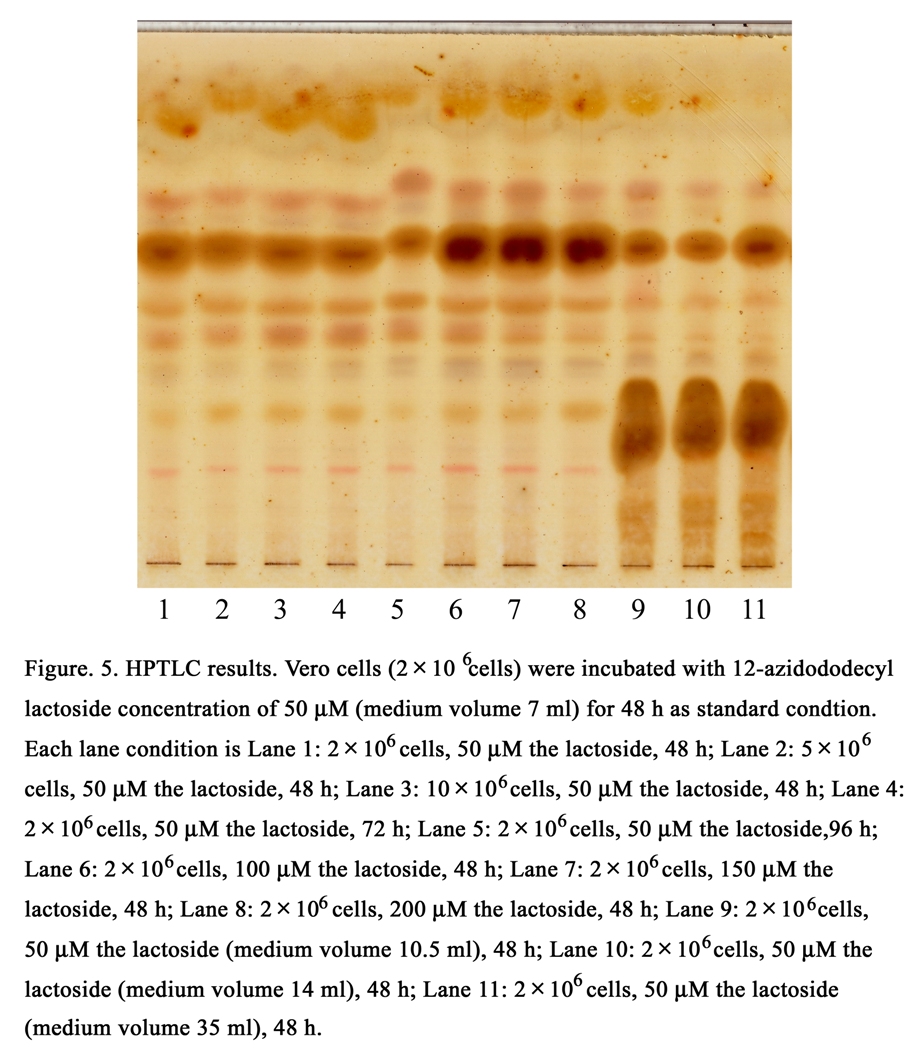

Supplement: Additional file 2 — HPTLC results. The data provided represent the production of the glycosides under optimized conditions. [file 1752-153X-1-26-S2.jpeg]
